# Supplementary material for: A Xeno-Free Protocol for Rapid Differentiation of Human iPSC-Derived Microglia from the KOLF2.1J Reference Line
Source: Bioengineering (Basel). 2025 Dec 30;13(1):45. doi: 10.3390/bioengineering13010045 (PMC12837815; doi:10.3390/bioengineering13010045)
Supplement: Supplementary file 1 [file bioengineering-13-00045-s001.zip › bioengineering-3988835-supplementary.pdf]

bioengineering

Technical Note

A Xeno-Free Protocol for Rapid Differentiation of Human iPSC-Derived Microglia from the KOLF2.1J Reference Line

Nélio A. J. Oliveira [1], Katherine R. Lewkowicz [1], Patricia A. Clow [1], Michael E. Ward [2], Mark R. Cookson [3], William C. Skarnes [1,*] and Justin A. McDonough [1,*]

[1] The Jackson Laboratory for Genomic Medicine, 10 Discovery Drive, Farmington, CT 06032, USA; nelio.oliveira@jax.org (N.A.J.O.); katherine.lewkowicz@jax.org (K.R.L.); patricia.clow@jax.org (P.A.C.)
[2] National Institute of Neurological Disorders and Stroke, National Institutes of Health, Bethesda, MD 20892 USA; michael.ward4@nih.gov
[3] Laboratory of Neurogenetics, National Institute on Aging, National Institutes of Health, Bethesda, MD 20892, USA; cookson@mail.nih.gov
* Correspondence: bill.skarnes@jax.org (W.C.S.); justin.mcdonough@jax.org (J.A.M.)

Supplemental Figures

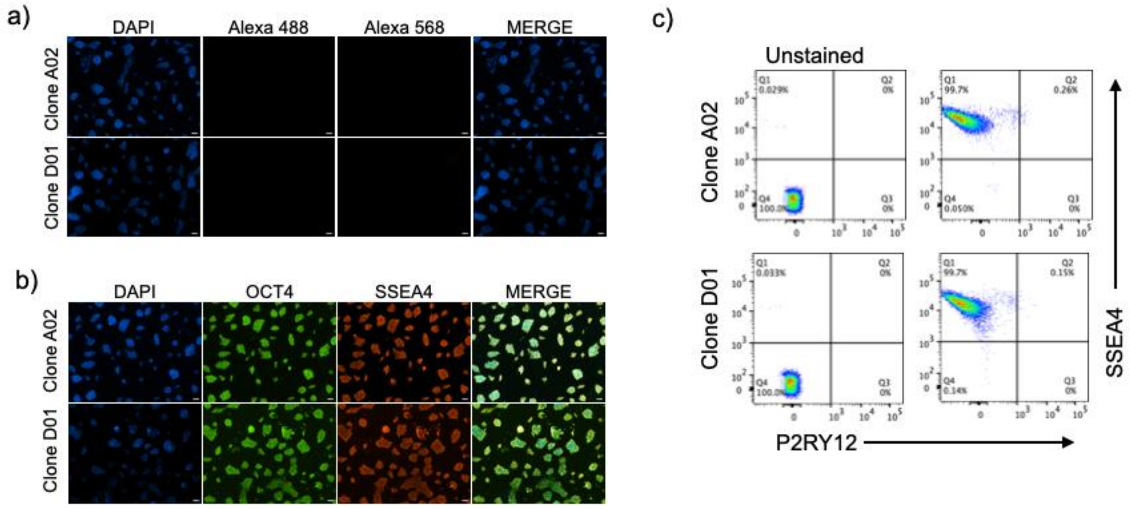

Supplemental Figure S1. Characterization of iMicroglia at the iPSC stage. A) Immunofluorescence (IF) for iPSC marker negative controls. B) Cells stained with OCT4 and SSEA4. C) Flow cytometry (FC) characterization using the iPSC marker SSEA4 and the microglia marker P2RY12.

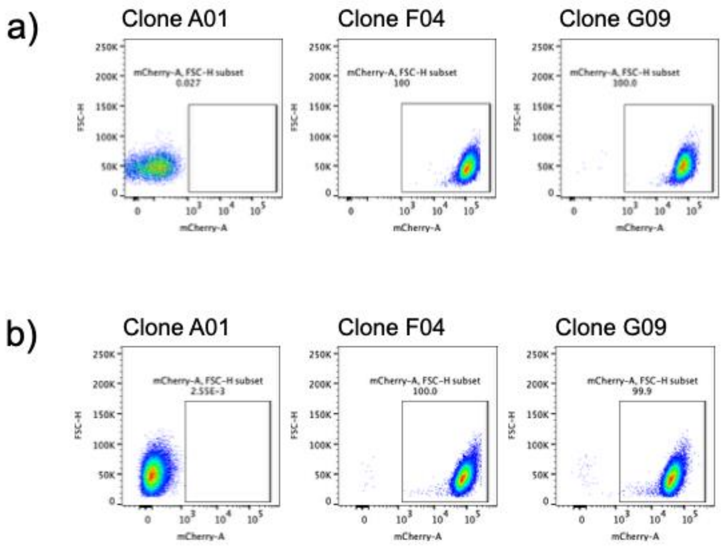

Supplemental Figure S2. Generation and validation of mCherry-expressing hiPSCs. (a) Flow cytometry analysis confirmed mCherry expression in SH4-2 knock-in clones F04 and G09. (b) Clones were expanded for two additional passages to verify stable mCherry expression. Clone A01, which lacked mCherry expression, was expanded as a negative control.

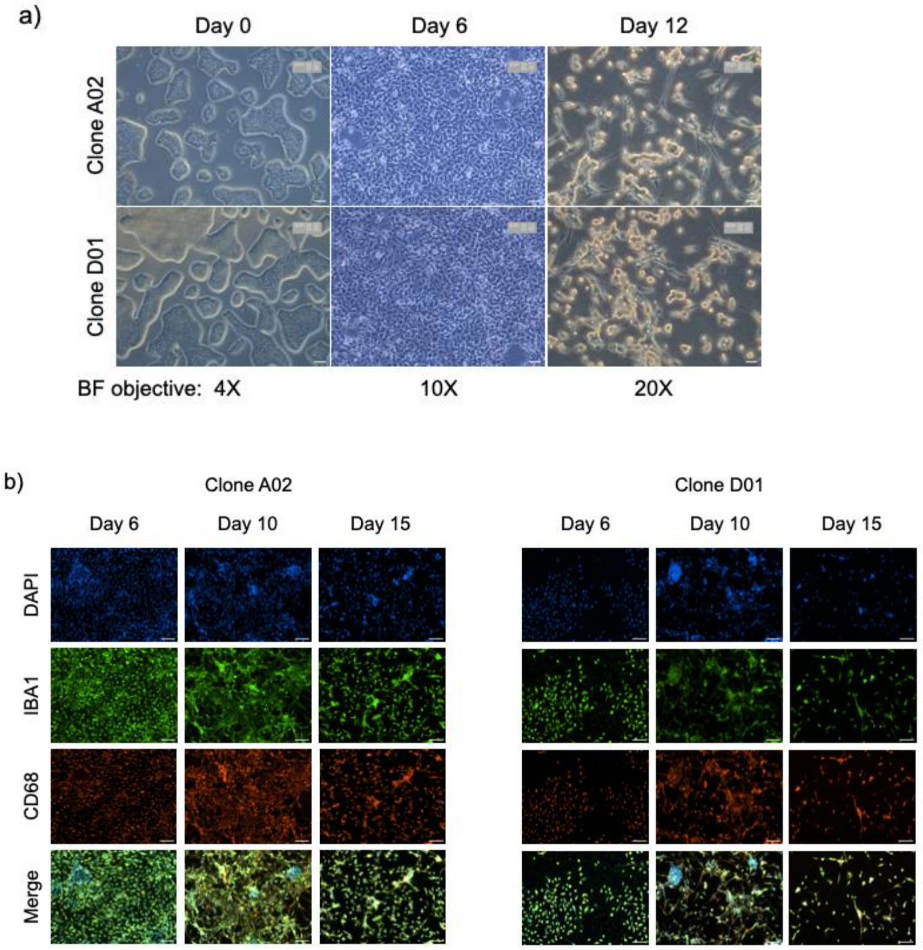

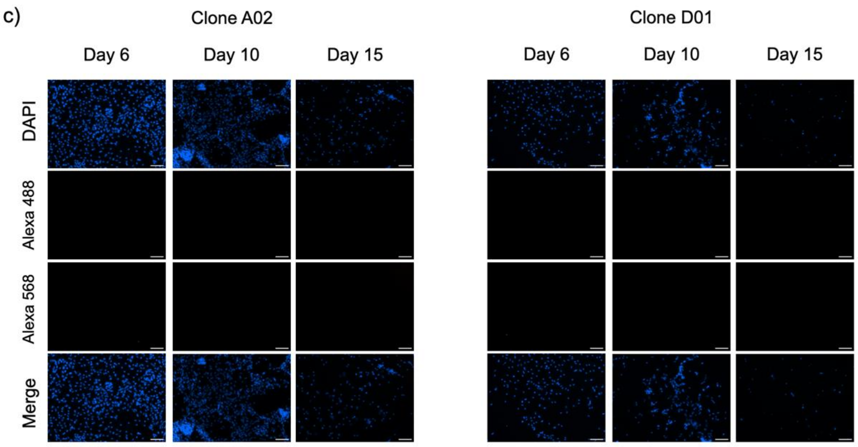

Supplemental Figure S3. Differentiation of i-Microglia on Poly-D-Lysine + Matrigel Substrate. (a) Brightfield (BF) images showing the progression from undifferentiated iPSCs at day 0 to day 15 cells exhibiting mature microglial morphology and (b,c) immunofluorescence (10× objective) showing microglial markers IBA1 and CD68. Secondary antibody only images are shown in panel c for AlexaFluor 488 and 568. Data shown for KOLF2.1J i-Microglia clones A02 and D01.

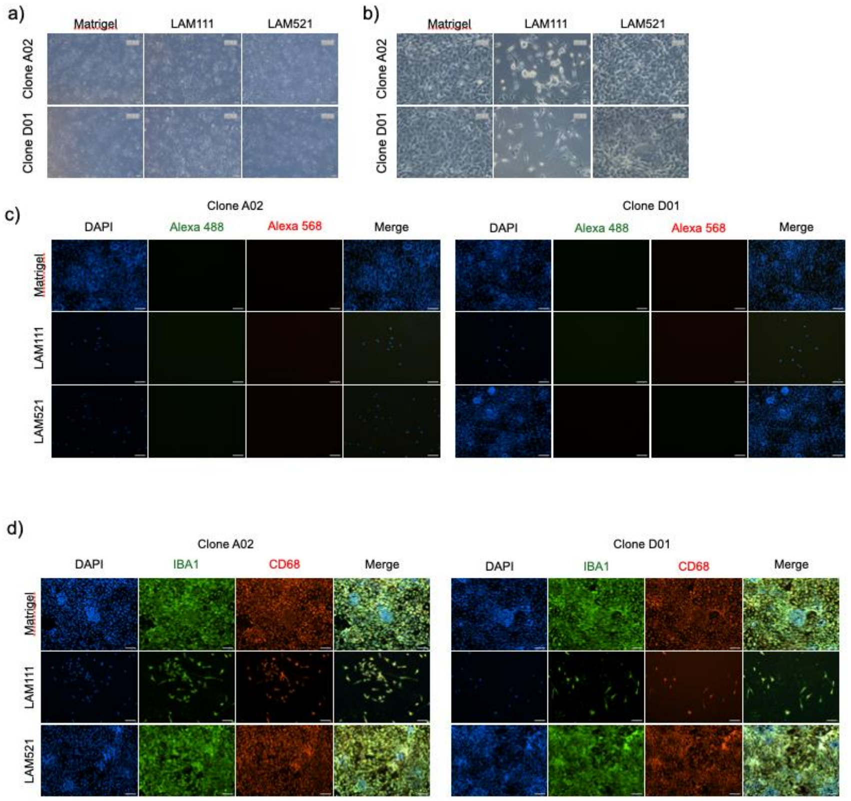

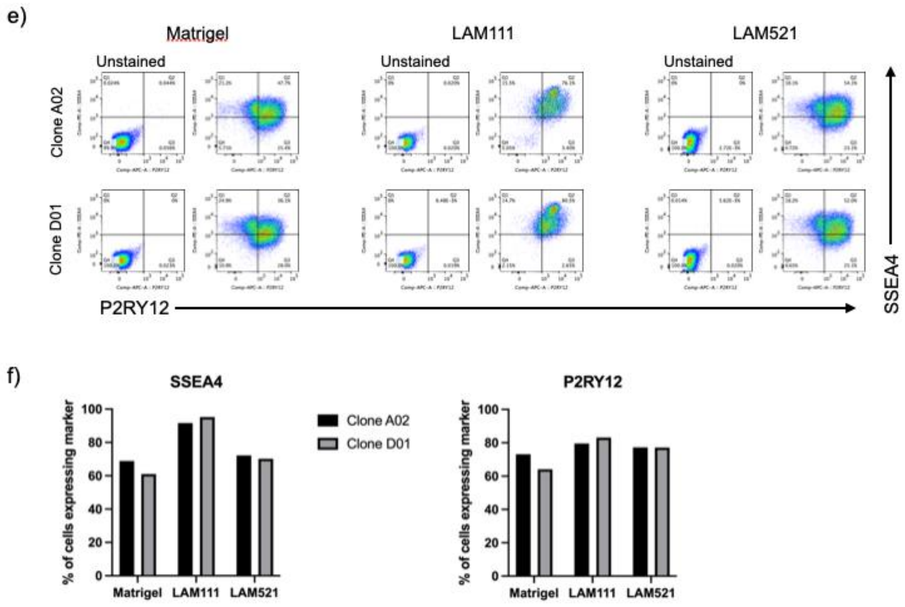

Supplemental Figure S4. Comparison of Substrate Conditions for i-Microglia Differentiation.(A–B) Representative brightfield images comparing differentiation on Matrigel, Laminin-111 (LAM111), and Laminin-521 (LAM521) at day 2 (A, 10× objective) and day 6 (B, 20× objective) for clones A02 and D01 (C) Negative control immunofluorescence (secondary antibody only) at day 6. (D) Immunofluorescence analysis at day 6 showing expression of IBA1 (green), CD68 (red), and DAPI (blue) in clones A02 and D01 across all three substrates. (E) Representative flow cytometry plots at day 6 showing SSEA4 (y-axis) and P2RY12 (x-axis) expression for each substrate condition in both clones. (F) Quantification of flow cytometry data showing the percentage of cells expressing SSEA4 (left) and P2RY12 (right) for clones A02 and D01 across Matrigel, LAM111, and LAM521 conditions.

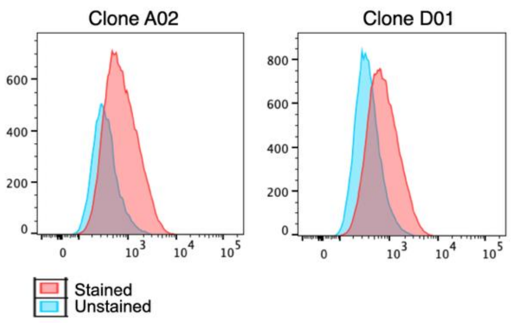

Supplemental Figure S5. Representative flow cytometry histograms showing CX3CR1 staining compared with matched unstained controls for i-Microglia clones A02 and D01.

Disclaimer/Publisher's Note: The statements, opinions and data contained in all publications are solely those of the individual author(s) and contributor(s) and not of MDPI and/or the editor(s). MDPI and/or the editor(s) disclaim responsibility for any injury to people or property resulting from any ideas, methods, instructions or products referred to in the content.

https://doi.org/10.3390/bioengineering13010045
